# Lessons from the Lab: SARS-CoV-2 Detection Rate amongst the Vaccinated Travel Lane and Non-Vaccinated Travel Lane Travellers into Malaysia

**DOI:** 10.21315/mjms2023.30.2.14

**Published:** 2023-04-18

**Authors:** Beh Chun Chuan, Nada Syazana Zulkufli, Chelvam Rajesvaran, Rohaizat Yon, Siow Loke Khee

**Affiliations:** 1 BP Clinical Lab Sdn Bhd, Temasya@Glenmarie, Selangor, Malaysia; 2 Lovy Pharmacy Sdn Bhd, Selangor, Malaysia

**Keywords:** COVID-19, SARS-CoV-2, travel medicine, border opening, pandemic travel

## Abstract

**Background:**

The vaccinated travel lane (VTL) between Malaysia and Singapore was implemented to facilitate travelling between countries without the need for quarantine.

**Objectives:**

i) Observe the rate of positive SARS-CoV-2 test results amongst inbound international travellers; ii) Explore possible factors associated with the outcome of test results between VTL and non-VTL travellers.

**Method:**

This is a retrospective cross-sectional study involving air travellers arriving in Malaysia via the Kuala Lumpur International Airport (KLIA) or Kuala Lumpur International Airport 2 (KLIA2) who were tested for SARS-CoV-2 by reverse transcriptase polymerase chain reaction (RT-PCR) from 29 November 2021 to 15 March 2022. Subject demographics and RT-PCR results were retrieved from the laboratory information system and statistically analysed.

**Results:**

Out of 118,902 travellers, mostly were Malaysian nationals (62.7%) and VTL travellers (68.2%) with a median age of 35 years old. A total of 699 (0.6%) of travellers tested positive on arrival, out of which 70.2% had cycle threshold (Ct) values > 30 (70.8% of VTL and 70.0% of non-VTL cohorts). Non-VTL travellers were 4.5 times more likely to test positive compared with VTL travellers (1.25% versus 0.28%; *P* < 0.001).

**Conclusion:**

Tighter entry requirements including vaccination status and testing frequency, the use of sensitive detection methods on arrival and similar public health policies between countries may have contributed to the VTL being a safe and cost-effective mode of travel.

## Introduction

The COVID-19 pandemic has significantly impacted healthcare and economic systems worldwide. Whilst travel restrictions have been shown to successfully contain the spread of disease by about 80% (1, 2), the severe impact it had on the economy and livelihoods called for it to be lifted but with strict compliance to risk mitigation strategies. According to the International Monetary Fund, the annual percent change in the real gross domestic product (GDP) growth of Malaysia dropped from 4.4% in 2019 to 5.6% in 2020 (3). Travel and tourism were among the most severely hit industries following movement restriction during the pandemic. What used to constitute 11.7% of the country’s GDP dwindled to 3.6% in 2020, with more than 62 million jobs in the travel and tourism sectors lost (4).

There had been significant heterogeneity in the border-entry strategies implemented by different countries, ranging from free entry without screening to screening of all passengers and entry prohibition of test positive travellers (5). On 29 November 2021, the vaccinated travel lane (VTL) was introduced between Malaysia and Singapore to allow fully vaccinated travellers from one country to enter into another by land or air without quarantine, subject to COVID-19 testing (6). It was made available to vaccinated adults as well as unvaccinated children below 12 years old who were accompanied by vaccinated adults. The VTL with Singapore was implemented in view of the similarities in COVID-19 management guidelines as well as high vaccination rates in both countries. As of the day of VTL initiation, at least 75.88% and 81.36% of the Malaysian and Singaporean populations, respectively, had been fully vaccinated (7, 8).

Vaccination rates vary across different countries. However, some countries, notably those from the African continent such as Nigeria and Kenya had < 10% population vaccination rates (9). In addition to vaccination rates, certain countries employed more relaxed COVID-19 management measures compared with Malaysia. For example, Switzerland, which has a high vaccination rate, has allowed tourist entry into the country without quarantine for vaccinated tourists since June 2021 (10). Although it is arguable that the high vaccination rate serves as a safety net, generally, vaccination does not equate to complete immunity from the virus, and the practice is not similar to that of Malaysia, which had adopted a more cautious approach toward reopening borders. Thus, the VTL served as a more balanced approach toward reopening the country.

Since the beginning of the VTL, BP Clinical Lab has been providing on arrival reverse transcriptase polymerase chain reaction (RT-PCR) testing for both VTL and non-VTL travellers at the Kuala Lumpur International Airport (KLIA) and Kuala Lumpur International Airport 2 (KLIA2). Due to the varied demographics of travellers and novelty of the VTL travelling concept, the authors undertook this study to observe the positivity rate of SARS-CoV-2 amongst inbound international travellers and explore possible factors associated with the outcome of test results between VTL and non-VTL travellers.

## Methods

### Study Design

This is a retrospective cross-sectional study involving all air travellers arriving in Malaysia via KLIA or KLIA2 who were tested for SARS-CoV-2 by RT-PCR in BP Clinical Lab, KLIA from 29 November 2021 to 15 March 2022. Data was collected by convenience sampling.

Subject demographics, nationality, country of departure, port of arrival, traveller type (VTL or non-VTL), sample number, date of test analysis, qualitative RT-PCR results and cycle threshold (Ct) values were retrieved from the laboratory information system (LIS) and exported to Microsoft Excel. Subjects with incomplete/invalid results, cancelled test requests, rejected samples and/or erroneous registrations were excluded from the analysis. Only Singapore-Malaysia VTL travellers were categorised as VTL travellers whereas travellers from other countries were categorised as non-VTL. Vaccination rates of a country were retrieved from international and local real-time databases and reflect the rates at the time of writing (7, 9).

### Measurement of Analytes

RT-PCR detects SARS-CoV-2 by employing primers that convert viral RNA into complementary DNA using the enzyme reverse transcriptase. In this study, two RT-PCR methods were used for the detection of SARS-CoV-2 nucleic acids i.e. real-time RT-PCR (rRT-PCR) and rapid RT-PCR. The method used is based on the passenger’s preference where rapid RT-PCR would be used for travellers requiring a report within 1.5 h at an extra cost and rRT-PCR for travellers requiring a report within 3 h–5 h.

rRT-PCR was performed using the Allplex™ SARS-CoV-2 Assay reagent (Seegene, Seoul) on the QuantStudio™ 5 Real-Time PCR system (ThermoFisher Scientific, Massachusetts). The presence of E and RdRp genes < 40 Ct value confer a positive test. Similar to rRT-PCR, rapid RT-PCR also detects SARS-CoV-2 by reverse transcription. However, all processes including nucleic acid lysis, amplification and data analysis are integrated into a single iPonatic Portable Molecule Workstation (Sansure BioTech, Changsha). The presence of N and ORF1ab genes < 40 Ct value confer a positive test.

For both methods, a negative test is reported when no target genes are detected; an inconclusive test is defined as the presence of only one gene; and an invalid test is defined as the failure of internal control amplification. All equipment had passed daily quality checks and correlation studies prior to clinical testing.

#### Data Analysis

Exported Microsoft Excel spreadsheet was transferred and analysed on IBM SPSS for Windows version 26.0 (Armonk, NY: IBM Corp). Data were evaluated for normality using the Kolmogorov-Smirnov test where normally distributed data were described as mean (standard deviation [SD]) values and non-parametric data as median (interquartile range [IQR]) values. Categorical data were expressed as frequency (*n*) and percentage (%).

Association between nominal categories were assessed using Chi-square test and a frequency chart of positive SARS-CoV-2 RT-PCR results against time was plotted to observe result trends. A *P*-value of < 0.05 is considered statistically significant.

## Results

A total of 118,937 data were primarily extracted from the LIS, 34 of which were excluded for departure travellers and one due to a cancelled test request. This results in a final total of 118,902 subjects and a response rate of 99.97%. Malaysians and VTL travellers formed the majority with a total cohort median age of 35 (IQR = 18.0) years old. A descriptive summary of the study cohort is provided in Table 1.

Non-VTL travellers were 4.5 times more likely to test positive for SARS-CoV-2 on arrival compared with VTL travellers with a *P*-value of < 0.001 (1.25% versus 0.28%). This consequently translates into a significantly higher percentage of positive RT-PCR results amongst travellers arriving in KLIA compared with those arriving in KLIA2 as only the latter caters to VTL travellers (*P* < 0.001) (Table 2).

Based on the detection of SARS-CoV-2 confirmatory gene, the majority of both the VTL and non-VTL groups had high Ct values (> 30) (Table 3). Overall, 70.2% of all travellers who tested positive for SARS-CoV-2 had Ct values >30.

**Figure 1:**
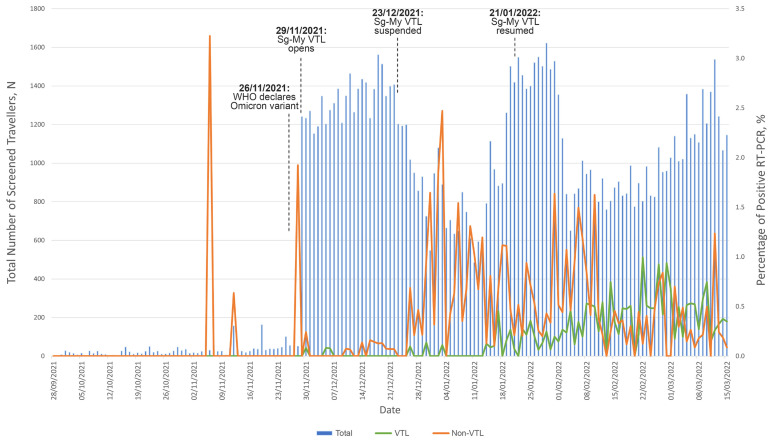
Trend of positive RT-PCR results amongst VTL and non-VTL travellers, and significant events throughout the study period Notes: WHO = World Health Organization; Sg-My VTL = Singapore-Malaysia Vaccinated Travel Lane

Figure 1 depicts the number of travellers screened and the percentage of travellers testing positive for SARS-CoV-2 RT-PCR test daily throughout the study period. The seeming surges in positive tests prior to 30 November 2021 were due to low passenger load during the first few months of border reopening.

## Discussion

At the time of writing, the majority of countries, including Malaysia, has transitioned to an endemic phase approach to manage COVID-19. By and large, restrictions that existed at the time of the VTL flights have been removed for international air travel. In our study, we found that travellers on the VTL flights had lower rates of SARS-CoV-2 positive test results on arrival compared with those of the non-VTL flights. We sought to look for significant factors associated with test result outcomes and deduce that tighter regulations and national vaccination policies contributed to the lower rate of SARS-CoV-2 positivity in VTL travellers.

VTL travellers are more tightly regulated in terms of higher frequency of testing and tighter entry requirements. Prior to departure and upon arrival, travellers are required to undergo RT-PCR testing for SARS-CoV-2. On arrival to KLIA and KLIA2, travellers are tested, contained in a supervised holding area, and are only allowed to leave for immigration clearance when the PCR test returns a negative result. Travellers who test positive are diverted to the relevant health authorities for further management. By containing travellers while waiting for test results, traveller movement is controlled and contact tracing is facilitated should a traveller test positive.

It is postulated that frequent testing especially by molecular-based methods and containment of travellers prior to the release of test results contributed to the success of the VTL in controlling the spread of infection across the Malaysia-Singapore border. Modelling studies have shown that testing and isolation of positive individuals on arrival reduces up to 91.7% of imported cases compared with allowing all the travellers entry without testing (5). According to Rosenberg and Holtgrave (11), more frequent testing including that of asymptomatic persons also reduces the epidemic burden. Additionally, testing and quarantining positive cases as opposed to quarantining all travellers saves money and optimises resources (5).

Effective 3 April 2020, international travellers were allowed entry to Malaysia upon observing a mandatory 14-day quarantine at select quarantine facilities. A total of 50% of the quarantine expenses were borne by the government for Malaysian citizens whereas foreigners were required to pay full charges not exceeding RM150 per person per day (12). However, travel quarantines are not cost-effective (5) and only benefit countries with near-zero prevalence in terms of within-country infection and hospitalisation rates (13). Large expenses on human resources, operational costs, contact tracing and procurement of medical supplies were incurred to run quarantine centres (14, 15), and simultaneous trade restrictions, labour shortage, and reduced industrial revenue resulted in fiscal stress (16). The test-and-hold strategy of the VTL was therefore considerably safe and cost-effective.

Traveller nationalities in the VTL population differ from that of the non-VTL population. VTL passengers were either Malaysian or Singaporean and non-VTL passengers were largely from other countries. A Canadian study found that disease incidence at the country of origin is a significant factor contributing to the positivity rate (17). Positive cases were lower in the VTL group probably due to similar public health policies and close collaboration in formulating policies between Malaysia and Singapore. Furthermore, both countries also have high vaccination rates and comparable social distancing and testing policies thus facilitating disease management across borders.

In line with recommendations and in order to decide on appropriate restrictions, countries should consider their COVID-19 incidence, local epidemic growth and travel (18). For example, during the rise of the Omicron variant, both countries bilaterally agreed to close the VTL on 23 December 2021 and subsequently reopened on 20 January 2022 when the wave of infections subsided (19). In contrast, non-VTL countries have different socioeconomic factors, which may affect the feasibility of implementing similar measures, at the cost of increasing disease transmission.

Another factor to consider is that vaccinated individuals who constitute the VTL group have lower rates of COVID-19 infection (20). Despite the emergence of new subvariants such as the BA.5 that may evade immune responses, vaccines have continued to demonstrate protection against severe illness resulting in hospitalisation and death (21, 22). In our study, we do not have data on the vaccination status of travellers in the non-VTL cohort; however, it is probable that the high vaccination rate of travellers contributed significantly to keeping numbers low in the VTL cohort. In a letter to the editor of the *Journal of Travel Medicine*, Torres (23) cited the 2021 Tokyo Olympics as a successful model of how high vaccination rates, frequent testing and social distancing helped curb the spread of COVID-19 during the games.

In terms of testing methods, PCR-based methods are more sensitive and specific compared with rapid antigen testing (RTK-Ag). PCR-based methods are especially useful in the testing of asymptomatic individuals and early infections where viral load is low—a strength that is lacking in RTK-Ag (24). In our study, a large fraction of travellers who tested positive on arrival had higher Ct values (Table 3). Considering a negative RT-PCR had already been ascertained prior to departure, a high Ct value on arrival may indicate early, asymptomatic infection. Identifying these individuals at the point of arrival pre-immigration clearance may deter the spread of disease into the community. However, RTK-Ag proves to be more economical and convenient with faster turn-around time. For this reason, RTK-Ag is favoured for mass testing be it in the tourism or industrial trade during the pandemic. For the VTL, only RT-PCR methods were used for on arrival testing, thus ensuring diagnostic accuracy. The availability of RT-PCR tests on site at KLIA further ensured rapid specimen processing and shorter holding time.

Limitations of this study includes its retrospective and single-centre nature. Non-VTL travellers who opted to get tested in the Ministry of Health in the airport were not included, possibly skewing the study’s findings on the non-VTL cohort. Additionally, genotyping of positive samples could not be performed due to the unavailability of the service.

## Conclusion

Non-VTL travellers were more likely to test positive for SARS-CoV-2 on arrival compared with VTL travellers. While testing of asymptomatic persons remains a controversial issue especially during the nation’s transition to endemicity, from a travel perspective, the VTL proved to be a necessary measure for a safe and economical border reopening. Following Malaysia’s full border opening on 1 April 2022, data monitoring of clinical testing, infectivity rates, and international travel should continue to aid us on ways to manage future pandemics as well as our response to crisis management.

## Figures and Tables

**Table 1:** Descriptive data of subjects (*N* = 118,902)

Parameter	Median [IQR]^a^	*n* (%)^b^
***Demographics***		
Age (years old)	35 [18.0]	
Nationality		
Malaysian		74,543 (62.7)
Singaporean		23,742 (20.0)
Others		20,617 (17.3)
***Travelling information***		
Port of entry		
KLIA		78,331 (65.9)
KLIA2		40,571 (34.1)
Traveller type		
VTL		81,059 (68.2)
Non-VTL		37,843 (31.8)
***RT-PCR result***		
Not detected		118,203 (99.4)
Detected (Ct value)	32.1 [5.61]	699 (0.6)
Ct ≥ 30		491 (70.2)
Ct < 30		208 (29.8)

Notes: IQR = interquartile range; KLIA = Kuala Lumpur International Airport; KLIA2 = Kuala Lumpur International Airport 2; VTL = vaccinated travel lane; Ct = cycle threshold;

a Continuous data presented as median [IQR];

b Categorical data presented as frequency (*n*) and percentage (%);

Percentage is calculated based on cohort total except Ct value which is calculated based on sub-group

**Table 2:** RT-PCR result distribution amongst screened international passengers arriving via KLIA and KLIA2 (*N* = 118,902)

Category	Detected (*n*)	Not detected (*n*)	Total (*n*)	Detected cases, %		*X*-statistic (df), *P*-value

				Of total (Category)	Of total (Cohort)	
***Port of entry***						
KLIA	583	77,748	78,331	0.74	0.49	96.08 (1),
KLIA2	116	40,455	40,571	0.29	0.10	< 0.001
***Passenger type***						
VTL	226	80,833	81,059	0.28	0.19	416.29 (1),
Non-VTL	473	37,370	37,843	1.25	0.40	< 0.001

Notes: KLIA = Kuala Lumpur International Airport; KLIA2 = Kuala Lumpur International Airport 2; VTL = vaccinated travel lane

**Table 3:** Distribution of passengers with positive RT-PCR result on arrival (*N* = 699)

Category	Ct value	Total (*n*)	Percentage of category
VTL	≥ 30	160	70.8
	< 30	66	29.2
Non-VTL	≥ 30	331	70.0
	< 30	142	30.0

Notes: VTL = vaccinated travel lane; Ct = cycle threshold

## References

[1] Chinazzi M, Davis JT, Ajelli M, Gioannini C, Litvinova M, Merler S (2020). The effect of travel restrictions on the spread of the 2019 novel coronavirus (COVID-19) outbreak. Science.

[2] Wells CR, Sah P, Moghadas SM, Pandey A, Shoukat A, Wang Y (2020). Impact of international travel and border control measures on the global spread of the novel 2019 coronavirus outbreak. Proc Natl Acad Sci U S A.

[3] International Monetary Fund (2022). IMF country information: Malaysia [Internet].

[4] World Travel and Tourism Council (2022). MALAYSIA 2022 Annual research: key highlights [Internet].

[5] Dickens BL, Koo JR, Lim JT, Sun H, Clapham HE, Wilder-Smith A (2020). Strategies at points of entry to reduce importation risk of COVID-19 cases and reopen travel. J Travel Med.

[6] MySafe Travel (2022). Frequently asked questions: vaccinated travel lane [Internet].

[7] COVIDNOW (2022). Population vaccinated, 2022 [Internet].

[8] Ministry of Health Singapore (2022). COVID-19 vaccination [Internet].

[9] Multilateral Leaders Task Force on COVID-19 (2022). Vaccine supply and delivery [Internet].

[10] Miller J (2021). Swiss accelerate reopening, allow large events with “COVID certificates” [Internet].

[11] Rosenberg ES, Holtgrave DR (2021). Widespread and frequent testing is essential to controlling coronavirus disease 2019 (COVID-19) in the United States. Clin Infect Dis.

[12] Malaysia National Disaster Management Agency (2020). Guidelines: entry and quarantine process Person Under Surveillance (PUS) arriving from abroad starting June 1, 2020.

[13] Wells CR, Pandey A, Fitzpatrick MC, Crystal WS, Singer BH, Moghadas SM (2022). Quarantine and testing strategies to ameliorate transmission due to travel during the COVID-19 pandemic: a modelling study. Lancet Reg Heal-Eur.

[14] Ministry of Finance Malaysia (2022). Estimated federal expenditure archives [Internet].

[15] Bernama (2021). Bajet 2022: Tambahan RM4 bilion untuk teruskan agenda tangani COVID-19 [Internet]. Am Bernama.com.

[16] Shang Y, Li H, Zhang R (2021). Effects of pandemic outbreak on economies: evidence from business history context. Front Public Health.

[17] Goel V, Bulir D, De Prophetis E, Jamil M, Rosella LC, Mertz D (2021). COVID-19 international border surveillance at Toronto’s Pearson Airport: a cohort study. BMJ Open.

[18] Russell TW, Wu JT, Clifford S, Edmunds WJ, Kucharski AJ, Jit M (2021). Effect of internationally imported cases on internal spread of COVID-19: a mathematical modelling study. Lancet Public Health.

[19] Bernama (2021). Decision to suspend ticket sales for VTL to curb Omicron variant [Internet]. *Nation.* New Straits Times.

[20] Haas EJ, Angulo FJ, McLaughlin JM, Anis E, Singer SR, Khan F (2021). Impact and effectiveness of mRNA BNT162b2 vaccine against SARS-CoV-2 infections and COVID-19 cases, hospitalisations, and deaths following a nationwide vaccination campaign in Israel: an observational study using national surveillance data. Lancet.

[21] Bowen JE, Addetia A, Dang HV, Stewart C, Brown JT, Sharkey WK (2022). Omicron spike function and neutralizing activity elicited by a comprehensive panel of vaccines. Science.

[22] Scobie HM, Johnson AG, Suthar AB, Severson R, Alden NB, Balter S (2021). Monitoring incidence of COVID-19 cases, hospitalizations, and deaths, by vaccination status-13 U.S. Jurisdictions, April 4–July 17, 2021. MMWR Morb Mortal Wkly Rep.

[23] Torres JR (2021). Are rapid antigen SARS-Cov-2 tests effective for mass screening of travelers at airports? The Olympic experience. J Travel Med.

[24] Fernandez-Montero A, Argemi J, Rodríguez JA, Ariño AH, Moreno-Galarraga L (2021). Validation of a rapid antigen test as a screening tool for SARS-CoV-2 infection in asymptomatic populations. Sensitivity, specificity and predictive values. eClinicalMedicine.

